# The PEGASUS Games: Physical Exam, Gross Anatomy, phySiology and UltraSound Games for Preclinical Medical Education

**DOI:** 10.24908/pocus.v6i1.14758

**Published:** 2021-04-22

**Authors:** Mary Hennekes, Sarah Rahman, Andrea Schlosser, Anne Drake, Tessa Nelson, Emily Hoffberg, Robert A Jones

**Affiliations:** 1 Case Western Reserve University School of Medicine Cleveland, OH; 2 The MetroHealth System Department of Emergency Medicine Cleveland, OH

**Keywords:** ultrasound, gamification, POCUS, preclinical, education

## Abstract

**Introduction: **Gamification engages learners and has successfully taught point-of-care ultrasound (POCUS) to residents and fellows. Yet ultrasound (US) curricula in undergraduate medical education remains limited. This study assessed a gamification model integrating US, anatomy, physiology, physical examination, and radiology created for preclinical medical students as compared with traditional didactic education. **Methods: **Twenty first-year medical students participated in a session on neck and thyroid material. Students were randomly assigned to a game or non-game group. Game students participated in games incorporating thyroid US with exam maneuvers, other imaging modalities, physiology, and pathology. Non-game students were taught the same material with an instructor. Students were assessed with a pretest and immediate and delayed post-tests. Group differences and scores were assessed using t-tests. A Likert scale evaluated learners’ opinions of the educational experience. **Results: **The game group performed better than the non-game group on the immediate post-test (p = 0.007, CI = [0.0305, ∞]). There was no significant difference between the groups on the delayed post-test (p = 0.726, CI = [-0.120, ∞]). Students in both groups felt more confident in their knowledge of the material, and all students in the game group agreed that the games encouraged teamwork. Most (9/10) stated the games allowed them to learn the material more effectively and would like to see more gamification (8/10). **Conclusion: **This US education model incorporating gamification for preclinical medical students promotes teamwork and is as effective for learning material than a traditional learning model. Students additionally convey a positive attitude towards gamification.

## Background

In recent years, point of care ultrasound (POCUS) has been increasingly incorporated into the bedside evaluation of patients by many different medical specialties. POCUS does not replace formal ultrasound (US) studies, but it can quickly provide information that may be life-saving for patients 1. Previous studies have demonstrated that intensivists and residents alike can be taught focused cardiac echocardiography with findings that consistently agree with those of experienced echocardiographers 2, 3. As POCUS increases in value as a safe and evidence-based tool, medical schools have begun to incorporate US into the physical examination, anatomy, and physiology curricula. Requirements for US education have been established at the graduate level, but integration of US into undergraduate medical education in the United States has varied across institutions 4. Some have successfully piloted US training as early as the preclinical years of medical education 5, 6, highlighting the applicability of US to physical examination and anatomy skills. Medical student response to US teaching has been enthusiastic with active student engagement 7, 8, and students express interest in pursuing US education 9, 10. 

Gamification, defined as an approach to teaching and learning where educators design more motivational learning experiences by using methods from game design in non-game contexts 11, 12, is an innovative method of instruction that increases learner engagement and aims to affect behaviors and learner attitudes. In a meta-analysis by Sailer and Homner, they reviewed the effect that gamification has had on different types of learning outcomes including cognitive, motivational, and behavioral learning as compared with conventional instructional methods and found a small but significant positive effect of gamification on each of these learning outcomes 13. They also found that when a component of social interaction was included, motivational and behavioral learning outcomes can be positively influenced by creating an environment of competition and collaboration. Within medical education, gamification has been successfully used to teach POCUS to residents and fellows, demonstrating an increase in short-term learning and overall satisfaction 14, 15; however, gamification itself remains an emerging and controversial area of education 12. Furthermore, when US education utilizing gamification is offered at events such as Ultrafest 16, students in all years of medical school are often required to complete the same games, although they have various levels of knowledge and clinical experience. 

Additionally, some medical schools have started to incorporate various diagnostic modalities with the physical exam and physiology during the preclinical years 17, 18. US, in conjunction with other diagnostic studies and concepts drawn from physiology, can be used to reinforce students' anatomical knowledge and hone diagnostic skills 5, 19. This study was designed to create a method of education that caters to the knowledge base of preclinical students (first- and second-year medical students) and utilizes game design taken from POCUS education as a vector. This study sought to determine the role of gamification in preclinical education and integration of clinical US, anatomy, physiology, physical examination, and radiology, to compare gamification with traditional education through both short- and long-term recall of material, and to promote self-guided education and teamwork.

## Methods

Twenty first-year medical students participated in a two-hour interactive session where they learned neck anatomy and radiology, reviewed thyroid physiology and pathology, and practiced physical exam maneuvers involving the neck as an adjunct to material already discussed in the medical school curriculum in the preceding weeks. All students were novices in POCUS having had less than 30 hours of prior US experience. Students were randomly assigned to a game or non-game group, and all students completed a pretest to gather demographic data, assess prior knowledge about neck and thyroid concepts, and determine if there were any group differences. This study was approved by the university’s Institutional Review Board. 

Both groups were taught US basics – anatomical directions, probe selection, and image optimization – through a short instructional video created by study investigators. Students randomized to the game group were then split into teams to complete 3 game stations. At each station, they watched a video created by study investigators relating to the station topic and participated in a self-guided game with time for topic review with a senior medical student following game completion. Students randomized to the non-game group were also split into 3 groups and watched the same videos but were taught the station material through a traditional lecture format. Instructors were senior medical students or residents with significant prior US experience. They were provided with an instructor manual and advised to not deviate from the script in order to standardize the learner experience. The same educational objectives were used for both the game and non-game stations. 

### Game Stations

The first game station was called “Ball Toss”. Ten plastic cups were arranged in a pyramid shape. Each cup corresponded to a numbered question about thyroid physiology, clinical vignettes, or sonographic images for identification of structures. Each team member took turns attempting to throw a ball from a specified distance into the cups. When they landed a ball in a cup, they received the question assigned to that cup. Team members then worked together to provide a correct answer to the question.

The second game station was an image “taboo” game in which students identified structures in the neck such as the thyroid gland, sternocleidomastoid muscle, and internal jugular vein in CT and MRI images as well as hands-on US scans. One student was asked to describe the structure without using any of the listed “taboo” words while the remaining team members named the correct structure and identified the structure on the image or using a handheld US device. For example, to identify the internal jugular vein on ultrasound, students could not use the words “carotid”, “jugular”, “vessel”, “MRI”, “X-Ray”, “ultrasound”, or “CT”.

During the third game station, “Pin the Tail on the M2”, students were timed while completing 3 physical exam maneuvers: palpating the thyroid gland, drawing a line for the location of a tracheostomy incision, and using color doppler with a handheld US to assess the carotid pulse. Accuracy of thyroid gland palpation was determined using handheld US. Students were timed continuously until the task was completed correctly. Five minutes were reserved at the end of each station to review game answers. Points were awarded for each game.

### Assessment and Analysis

Following completion of the interactive session, both groups took one version of two 15-question multiple choice post-tests that assessed the anatomy, physiology, physical exam, radiology, and US topics taught during the session. To ensure similar difficulty between the two post-test versions, medical students in their clinical years volunteered to complete both post-tests prior to the study session. A paired t-test was used to analyze the students’ differences in exam scores between post-test A and post-test B and demonstrated no difference between the two (p = 0.5467). The game group initially received post-test A, and the non-game group received post-test B. Two weeks following the session, both groups took a second post-test – the game group completed post-test B while the non-game group completed post-test A. Due to a mistake in post-test B, question #4 was thrown out of analysis. Three reminder emails were sent to all students to encourage completion of the second post-test. 

Group demographics were analyzed descriptively. Group differences in pre- and post-test scores were assessed using paired t-tests as well as covariant analyses adjusting for prior US experience. Student experience was evaluated using Likert scales that was then assigned a numerical value for analysis (strongly disagree = 1, strongly agree = 5). Analysis was performed using Jamovi (The jamovi project (2019). jamovi (Version 0.9)).

## Results

Demographics and prior US experience of the student in both the game and non-game group are depicted in Table 1. 

**Table 1:** Group Characteristics

**Characteristic**	**Game Group**	**Non-game Group**
Gender, n		
Male	8	6
Female	2	3
Prefer not to say	0	1
Age, mean y	25.3y	23.4y
Degree, n		
Biology	2	5
Neuroscience	2	0
Biochemistry	2	2
Engineering	1	1
Other STEM degree	3	2
Prior healthcare job/volunteering, n		
Yes	9	5
No	1	5
Ultrasound experience, mean hr*	1.9hr	4.65hr
*Most students (19/20) had <10 hours of previous ultrasound experience; 1 student in the non-game group reported 30 hours (p = 0.395)		

There were 10 students in each group. The majority of students in both groups were male and had completed a degree related to science or healthcare. Most (19/20) students had less than 10 hours of prior US experience. One student listed 30 hours of prior US experience. Average US experience in the game group was 1.9 hours and 4.65 hours in the non-game group (p = 0.395). 

Detailed information about individual student pretest and post-test scores can be found in Table 2. 

**Table 2:** Student Pretest and Post-test Scores

**Group**	**Prior Ultrasound Experience (hr)**	**Pretest Score (%)**	Immediate Post-test Score (%)*	Delayed Post-test Score (%)*
**Game group **				
Student 1	1.5	60.0%	93.3%	85.7%
Student 2	3.0	80.0%	86.7%	78.6%
Student 3	1.0	80.0%	93.3%	85.7%
Student 4	10.0	60.0%	93.3%	100.0%
Student 5	1.5	80.0%	100.0%	71.4%
Student 6	1.0	60.0%	86.7%	78.6%
Student 7	1.5	60.0%	100.0%	71.4%
Student 8	0.0	60.0%	100.0%	85.7%
Student 9	0.0	60.0%	100.0%	92.9%
Student 10	0.5	40.0%	93.3%	85.7%
**Mean, SD**	**2.0, 2.9**	**64.0%, 12.6%**	**94.7%, 5.6%**	**86.4%, 8.9%**
**Non-game group **				
Student 1	1.0	40.0%	78.6%	86.7%
Student 2	0.5	40.0%	85.7%	86.7%
Student 3	2.0	60.0%	92.9%	93.3%
Student 4	0.0	60.0%	78.6%	78.6%
Student 5	6.0	80.0%	92.9%	100.0%
Student 6	3.0	40.0%	78.6%	Did not complete
Student 7	30.0	60.0%	100.0%	Did not complete
Student 8	2.0	40.0%	78.6%	73.3%
Student 9	1.0	100.0%	92.9%	93.3%
Student 10	1.0	60.0%	85.7%	86.7%
**Mean, SD**	**4.65, 9.1**	**58.0%, 19.9%**	**83.6%, 7.9%**	**87.3%, 8.5%**
* The game group took post-test A immediately following the session and post-test B 2 weeks following the session. The non-game group took post-test B immediately following the session and post-test A 2 weeks later.				

There were no statistically significant group differences on the pretest when comparing prior knowledge of the session topics between two groups (p = 0.4313, CI = [-0.0966, 0.217]). The game group scored higher than the non-game group on the post-test immediately following the session (p = 0.007, CI = [0.0305, ∞]). There was no significant difference between the groups’ performances on the delayed post-test (p = 0.810, CI = [-0.110, ∞]). When controlling for prior US experience, the game group still did significantly better than the non-game group (p = 0.003) on the immediate post-test while there was still no difference between two groups on the delayed post-test (p = 0.265). Of note, all 10 students in the game group completed the second post-test while only 8 students in the non-game group completed it. 

**Figure 1:**
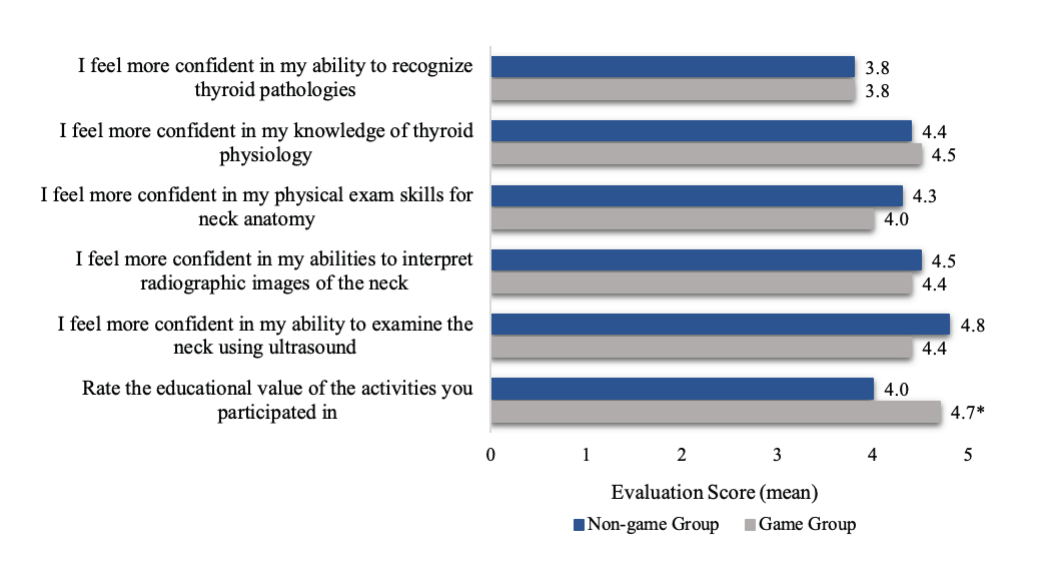
Student evaluations by group. Questions evaluating confidence used a Likert scale. A score of 1 corresponds to an answer of “strongly disagree” and a score of 5 corresponds to an answer of “strongly agree”. The question rating educational value used a scale in which 1 corresponded to “no value” to 5 which corresponded to “highest value”. *The game group rated the educational value of their activities significantly higher than that of the non-game group (p = 0.010)

Students in both groups rated their confidence of their knowledge of the material higher following the session. When asked to rate the educational value of the activities the students participated in, those in the game group reported a higher educational value than those in the non-game group (p = 0.010, CI = [0.217, 1.18]). Figure 1 depicts the questions asked of students in both groups regarding overall satisfaction and compares the average response of each group. 

All (10/10) of the students in the game group agreed or strongly agreed that the games encouraged teamwork. Most (9/10) felt the games taught the material more effectively than didactic educational methods and did not hinder their learning of the material. Most (8/10) also stated that they would like to see more gamification in their medical school curriculum.

## Discussion

The preclinical years of medical education are undergoing rapid innovation as curricula shifts away from the classroom and towards online learning 20. Nationally, nearly 25% of second-year medical students reported they almost never attended class during their preclinical years 21. This educational model threatens important components of preclinical education such as hands-on physical exam techniques and diagnostic methods and undermines continued reinforcement of teamwork amongst peers. The ability to work on a team is essential for effective and efficient healthcare delivery. Creating a curriculum that integrates many clinical disciplines can help preclinical medical students synthesize concepts and better prepare for the wards. For example, Alerhand et al. demonstrated that it was feasible to create a POCUS curriculum which integrated anatomy, pathophysiology, and the physical exam for first-year medical students in their renal course and that students found it increased confidence in their understanding of the material 22. This study similarly demonstrated that the integration of US, radiology, physical exam, and physiology is effective as a learning adjunct for preclinical medical students and also promotes teamwork amongst them.

Unlike in Canada where The Canadian Ultrasound Consensus for Undergraduate Medical Education Group has recommended 85 US curricular elements for inclusion in all Canadian medical schools including the use of peer teachers, small-group scanning, simulation, and interprofessional training, medical education in the United States has no such consensus 23, 24, 25. POCUS, particularly when taught using hand-held portable US devices, is well-suited to teaching US as an adjunct to the preclinical medical school curriculum. It can be incorporated easily through the use of trained senior medical student peer instructors^26^ or interprofessional near-peers 27, especially when access to expert faculty is limited. While US education in the United States has no standardization, this study provides additional evidence to the increasingly growing body of research that medical students desire an US education prior to graduation 7, 8, 10. 

Using gamification in this study increased student confidence, teamwork, autonomy, and motivation to learn by allowing for active learning to promote material retention. Yet the influence of gamification in education remains controversial 12. Proponents of gamification in education believe the goal of gamification is to encourage an environment that supports learning and productive social interactions, and students seem to perceive this educational environment positively. On the other hand, gamification can be complex and competitive, something that may cater predominantly to learners who are proactive and intrinsically motivated. 

Other gamification models used in POCUS education have demonstrated a positive cognitive and behavioral effect for learners. Students in the SonoSlam national medical student US competition^28^ and SonoGames resident competition^29^ reported increased confidence and clinical performance. An US game to enhance learning of gastrointestinal anatomy and physiology for first-year medical students by measuring blood vessel velocity was successful in increasing student interaction 30. This study, too, found that a game model is effective for material retention, at least in the short-term, increases student confidence in the material, and is positively received. But like prior studies, this study failed to demonstrate any differences in long-term retention of the material when comparing student knowledge for those who participated in the games with those who received traditional didactic education. 

There is no one right way to incorporate POCUS into medical education. Students prefer small-group educational sessions as compared with large groups 31. They also report that hands-on practice and the use of video clips in contrast to lectures and still images are more practical for learning 31. The use of portfolios in higher education has also been shown to foster an improvement in knowledge and understanding of material, increase personal responsibility for learning, emphasize reflection and engagement^32^, ^33^ and may be an additional adjunct to POCUS education. This study adds to the body of literature that demonstrates that gamification is a feasible and well-received method to incorporate POCUS into medical education. 

## Limitations

The major limitation of this study is the small sample size, though the strength of the study comes from being the first study to compare a group of students who participated in US games to those who participated in a traditional didactic format. The scope of this study was also narrow, as only thyroid and neck material were covered. Results of this study might not hold true for gamification models encompassing other organ systems or for POCUS education as a whole.

Additionally, the material covered in the session aligned with the information students were learning during regular curricular lectures and small group learning. Between the post-test immediately following the session and the delayed post-test, students may have studied the information in variable amounts, affecting retention of the material due to the session education alone. Selection bias may have also influenced results. It is conceivable that if the non-responders had completed the delayed post-test, this may have influenced the outcome.

## Conclusions

This ultrasound education model incorporating gamification for preclinical medical students promotes teamwork amongst peers and is as effective, if not more effective, for learning material and retaining knowledge than a traditional learning model. Students appear to react positively towards gamification and feel that it improves their learning and understanding of concepts. Future research should continue to test the use of gamification and US to teach other organ systems and at other stages of medical education. Future studies should also create new games to compare effectiveness of different applications of gamification to education. Although our study did not find an impact of gamification on long-term material retention, future trials should continue to assess if gamification increases information retention in different settings.

## Disclosures

The study authors have no disclosures or conflicts of interest to report.

## Statement of Ethics Approval/Consent

All subjects who participated in this study gave informed consent. This study was approved by the IRB at Case Western Reserve University.

## Supplementary Material 

Supplemental Information Document S1
