# Supplementary material for: The PEGASUS Games: Physical Exam, Gross Anatomy, phySiology and UltraSound Games for Preclinical Medical Education
Source: POCUS J. 2021 Apr 22;6(1):22–8. doi: 10.24908/pocus.v6i1.14758 (PMC9979934; doi:10.24908/pocus.v6i1.14758)
Supplement: Supplemental Information Document S1 [file pocusj-06-14758-s001.pdf]

Supplemental Information:

Pretest
To create your unique identifier, use the last 3 digits of your phone number and the first 3 digits of your childhood street. For example, 784LAK.

Unique identifier: ____________

Demographics:
Please specify your gender.

Male Female Prefer not to say Prefer to self-describe ____________

What is your age? ____

Please specify your race.

White African American Native American Asian/Pacific Islander

Other (please specify) ___________________

Please specify your ethnicity.

Hispanic Non-Hispanic

List your most recent undergraduate major and/or postgraduate degree prior to attending medical school:
____

Describe any healthcare-related work experience prior to attending medical school:
____

Estimated hours of previous ultrasound experience: ____

Assessment
1. Identify this ultrasound probe.

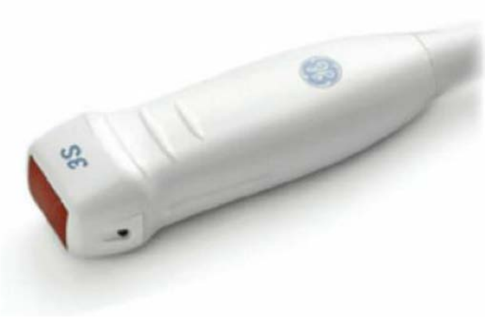

a. Phased array
b. Curvilinear
c. Linear
d. Phased linear
e. Phased curvilinear

2. If a patient with the high TSH and low T3/T4 presents to your clinic, what is the most likely diagnosis?
a. Primary hypothyroidism
b. Secondary hypothyroidism
c. Central hypothyroidism

d. Hyperthyroidism

3. What molecules combine to form T3 (thyroxine) while attached to thyroglobulin?

a. MIT + MIT
b. DIT + DIT
c. MIT + DIT
d. Iodine + DIT
e. Iodine + MIT

4. To palpate the carotid pulse, move your fingers posterolaterally from....

a. The sternocleidomastoid muscle
b. The sternal notch
c. The thyroid gland
d. The thyroid cartilage

5. Which of the following imaging modalities correctly describes this image?

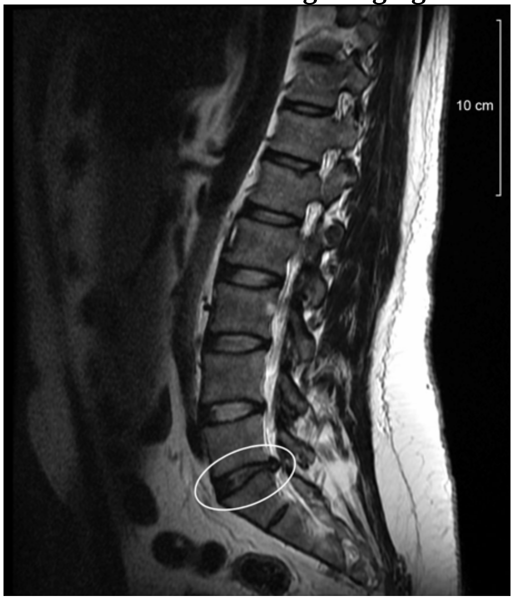

a. Magnetic resonance imaging (MRI)
b. Computed tomography (CT)
c. Ultrasound
d. X-ray

Post-test Version A:
To create your unique identifier, use the last 3 digits of your phone number and the first 3 digits of your childhood street. For example, 784LAK.

Unique identifier: ____________

<u>Assessment:</u>

1. The thyroid is immediately inferior to the…

a. Cricoid cartilage
b. Manubrium
c. Thyroid cartilage
d. Hyoid bone
e. Sternal angle

2. Assuming the probe is in the proper position, the right side of this image represents…

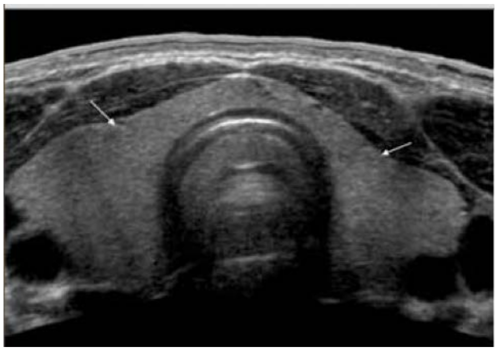

a. Patient's right
b. Patient's left
c. Patient's feet
d. Patient's head

3. Where is the carotid pulse best palpated?

a. Between the thyroid gland and sternocleidomastoid muscle
b. Between the cricoid cartilage and anterior scalene muscle
c. Between the thyroid cartilage and sternocleidomastoid muscle
d. Between the thyroid gland and anterior scalene muscle

4. Which maneuver is utilized to increase the size of the internal jugular vein?

a. Left lateral decubitus position
b. Valsalva
c. Squatting
d. Rapid standing

5. Indicate which of the following is a classic symptom of hyperthyroidism.

a. Dry skin
b. Heat intolerance
c. Joint dislocations
d. Unintentional weight gain
e. Constipation

6. In this image, which letter indicates the carotid artery?

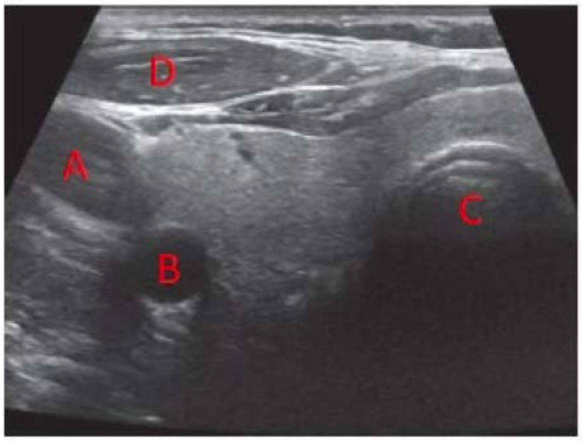

a. A
b. B
c. C
d. D

7. In secondary hypothyroidism, what changes in the serum TSH would you expect?
a. Increased
b. Decreased
c. No change

8. If a patient with low TSH presents to your clinic, which of the following should NOT be on your differential diagnosis?
a. Hyperthyroidism
b. Central (tertiary) hypothyroidism
c. Primary hypothyroidism
d. Secondary hypothyroidism

9. Identify this probe.

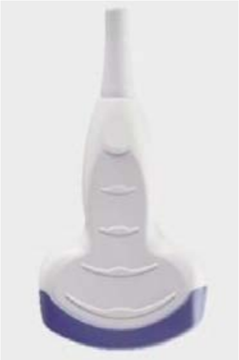

a. Linear
b. Phased array
c. Curvilinear
d. Phased curvilinear

10. Which ultrasound probe would be used for a superficial neck examination?
a. Phased array

b. Linear
c. Curvilinear

11. Name this structure.

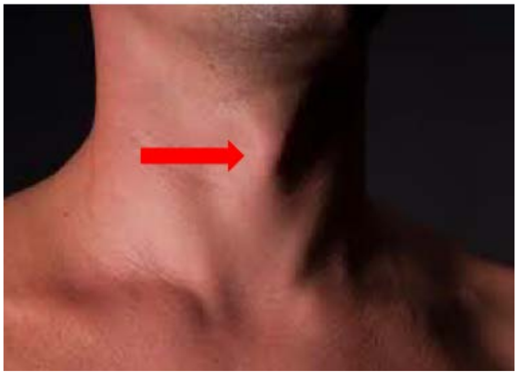

a. Thyroid gland
b. Cricoid cartilage
c. Thyroid cartilage
d. Sternocleidomastoid muscle

12. What are the structures contained within the carotid sheath?

a. Phrenic nerve, carotid artery, internal jugular vein
b. Vagus nerve, carotid artery, internal jugular vein
c. Vagus nerve, vertebral artery, internal jugular vein
d. Vagus nerve, carotid artery, external jugular vein
e. Vagus nerve, carotid vein, internal jugular vein

13. What three steps does thyroid peroxidase catalyze during thyroid hormone synthesis?

a. T3/T4 hydrolysis, iodine oxidation, organification
b. Deiodination, organification, coupling
c. Iodide transport, iodide oxidation, deiodination
d. Iodine oxidation, organification, coupling

14. The orientation of this image can be described as…

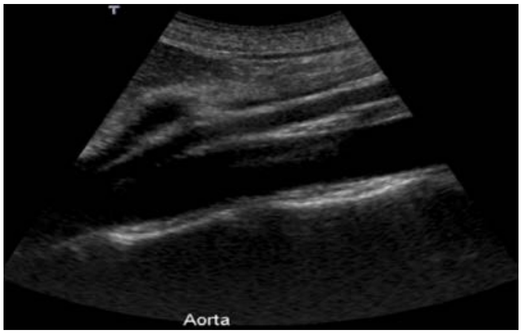

a. Oblique
b. Transverse
c. Horizontal
d. Sagittal

15. What is the structure indicated by the red box?

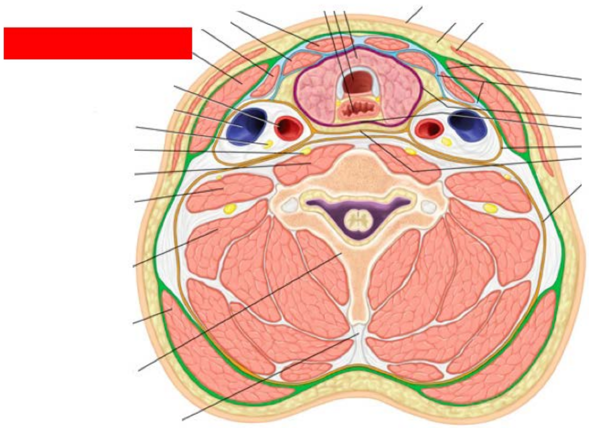

Copyright © 2009 Wolters Kluwer Health | Lippincott Williams & Wilkins

7-10 Cervical Fascia, B. Cross Section

a. Sternocleidomastoid muscle
b. Trapezius muscle
c. Thyroid gland
d. Platysma

Post-test Version B:
To create your unique identifier, use the last 3 digits of your phone number and the first 3 digits of your childhood street. For example, 784LAK.

Unique identifier: ____________

Assessment:

1. Which of the following is listed correctly from superior to inferior?
a. Thyroid cartilage, cricoid cartilage, thyroid
b. Cricoid cartilage, thyroid cartilage, thyroid
c. Thyroid cartilage, thyroid, cricoid cartilage
d. Cricoid cartilage, thyroid, cricoid cartilage
e. Thyroid, cricoid cartilage, thyroid cartilage

2. Assuming the probe is in the proper position, the left side of this image represents…

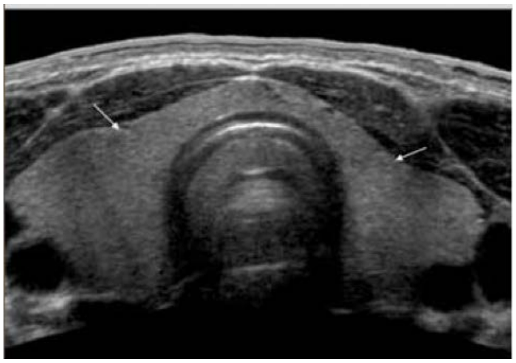

a. Patient’s right
b. Patient’s left
c. Patient’s feet
d. Patient’s head

To palpate the carotid pulse, move your fingers anteromedially from...
a. The sternocleidomastoid muscle
b. The sternal notch
c. The thyroid gland
d. The thyroid cartilage

4. What is the proper positioning of the physician during a thyroid exam?
a. Directly in front of the patient
b. To the right side of the patient
c. Above the patient
d. Directly behind the patient

5. Indicate which of the following is not a classic symptom of hypothyroidism?
a. Constipation
b. Cold intolerance
c. Moist skin
d. Weight loss
e. Anxiety

6. In this image, which letter indicates the internal jugular vein?

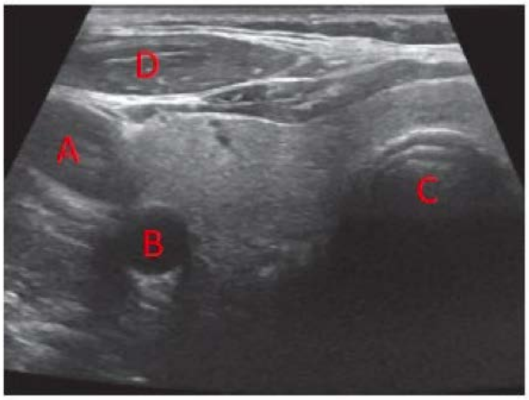

a. A
b. B
c. C
d. D

7. In primary hyperthyroidism, what changes in serum TSH value would you expect?

a. Increased
b. Decreased
c. No change

8. If a patient with high TRH, low TSH, and low T3/T4 presents to your clinic, what is the most likely diagnosis?

a. Hyperthyroidism
b. Central (tertiary) hypothyroidism
c. Primary hypothyroidism
d. Secondary hypothyroidism

9. Identify this probe.

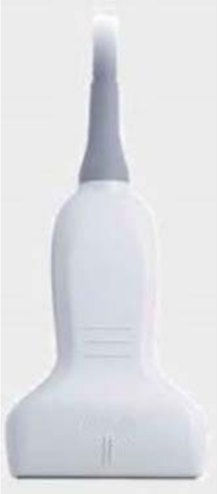

a. Linear
b. Phased array
c. Curvilinear
d. Phased curvilinear

10. Which ultrasound probe would be useful for an abdominal exam?

a. Curvilinear

b. Phased array
c. Linear

11. The isthmus of the thyroid overlies which portion of the trachea?

a. First and second rings
b. Second and third rings
c. Third and fourth rings
d. Fourth and fifth rings

12. Which of the following is not found within the carotid sheath?

a. Phrenic nerve
b. Internal jugular vein
c. Carotid artery
d. Vagus nerve

13. What enzyme catalyzes T4 to T3 conversion in target tissues?

a. Tryptase
b. Thyroid peroxidase
c. Lysosomal enzymes
d. 5'-deiodinase

14. The orientation of this image can be described as…

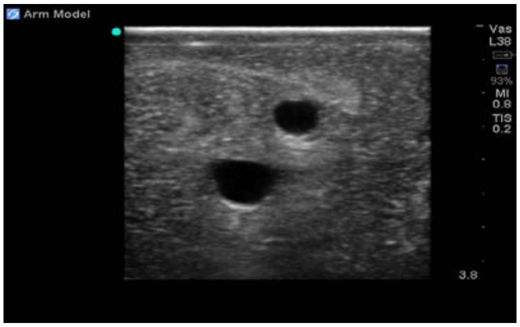

a. Sagittal
b. Coronal
c. Oblique
d. Transverse

15. What is the structure indicated by the blue box?

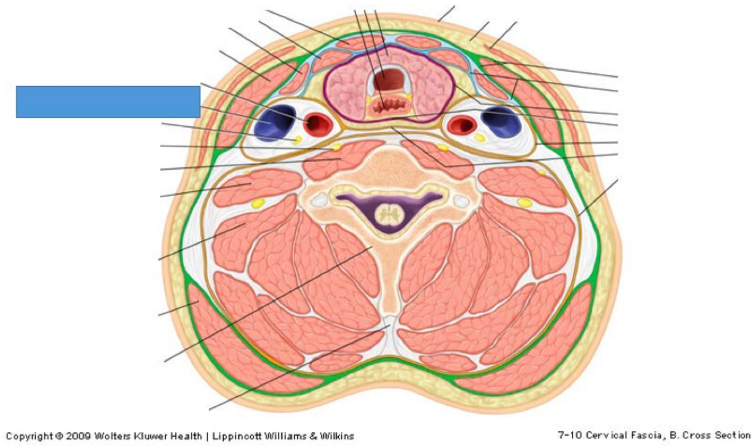

a. External jugular vein
b. Carotid vein
c. Internal jugular vein
d. Brachiocephalic vein

Study Evaluation:
To create your unique identifier, use the last 3 digits of your phone number and the first 3 digits of your childhood street. For example, 784LAK.

Unique identifier: ____________

Rate the educational value of the activities you've participated in on a scale of 1 to 5 (1 = no value, 5 = highest value).
1 2 3 4 5

I feel more confident in my ability to ultrasound the neck.
Strongly disagree Disagree Neutral Agree Strongly agree

I feel more confident in my ability to interpret radiographic images of the neck.
Strongly disagree Disagree Neutral Agree Strongly agree

I feel more confident in performing physical exam maneuvers involving the neck.
Strongly disagree Disagree Neutral Agree Strongly agree

I feel more confident in understanding thyroid physiology.
Strongly disagree Disagree Neutral Agree Strongly agree

I feel more confident in my ability to recognize and diagnose thyroid pathology.
Strongly disagree Disagree Neutral Agree Strongly agree

Only answer the following questions if you were in the gamification group (group 1).

The games encouraged teamwork.
Strongly disagree Disagree Neutral Agree Strongly agree

Participating in the games more effectively taught me the material.
Strongly disagree Disagree Neutral Agree Strongly agree

The games hindered my ability to learn the material.
Strongly disagree Disagree Neutral Agree Strongly agree

The games created conflict and stress amongst my teammates.
Strongly disagree Disagree Neutral Agree Strongly agree

I would like to see gamification integrated into the medical school curriculum.
Strongly disagree Disagree Neutral Agree Strongly agree
